# A rare c.183_187dupCTCAC mutation of the acetylcholine receptor *CHRNE* gene in a South Asian female with congenital myasthenic syndrome: a case report

**DOI:** 10.1186/s12883-016-0716-y

**Published:** 2016-10-07

**Authors:** Thashi Chang, Judith Cossins, David Beeson

**Affiliations:** 1 Department of Clinical Medicine, Faculty of Medicine, University of Colombo, 25, Kynsey Road, Colombo 08, Sri Lanka; 2 Neuromuscular Disorders Group, Nuffield Department of Clinical Neurosciences, Weatherall Institute of Molecular Medicine, Oxford, OX3 9DS, United Kingdom

**Keywords:** Congenital myasthenic syndrome, CHRNE, c.183_187dupCTCAC

## Abstract

**Background:**

Congenital myasthenic syndromes (CMSs) occur as a result of genetic mutations that cause aberrations in structure and/or function of proteins involved in neuromuscular transmission. Acetylcholine receptor epsilon (ε) subunit (*CHRNE*) gene mutations account for about 30–50 % of genetically diagnosed cases. We report a rare *CHRNE* gene mutation in a South Asian female with CMS.

**Case presentation:**

A 17-year-old Maldivian female presented with bilateral partial ptosis, fatigable proximal muscle weakness and slurring of speech noted since the age of 2 years. She could not run, had difficulty negotiating stairs and rising from a seated position, and fatigues when speaking at length. Her birth and past medical histories were otherwise unremarkable. There is no parental consanguinity or family history of muscle disorders.

On examination, she had a BMI of 18 kg/m^2^, bilateral fatigable partial ptosis, complete external ophthalmoplegia and fatigable proximal muscle weakness (MRC grade 4/5). Apart from spinal scoliosis the rest of the examination was normal.

Haematological and biochemical investigations including serum lactate level and thyroid functions were normal. Acetylcholine receptor antibodies and muscle specific kinase antibodies were not detected in serum. Repetitive nerve stimulation showed marked decrement (>30 %) in nerve-muscle pairs in the face and forearm. Her DNA sequencing revealed a c.183-187dupCTCAC mutation in *CHRNE*.

She remained functionally independent on pyridostigmine treatment.

**Conclusions:**

This case describes a rare mutation of the *CHRNE* gene in CMS and highlights the relevance of genetic diagnosis in CMS. It further adds to map the occurrence of such mutations in Asian populations.

## Background

Congenital myasthenic syndromes (CMSs) are a heterogeneous group of genetically determined neuromuscular transmission disorders characterized by fatigable muscle weakness that usually manifest early in life. Genetic mutations alter structural and functional proteins at the motor endplate leading to impaired neuromuscular transmission. To date, at least 22 CMS-associated disease genes have been identified, of which acetylcholine receptor deficiency due to ε subunit (*CHRNE*) gene mutations have been found to be the commonest [1–3]. However, CMSs are rare and the mutation c.183_187dupCTCAC in *CHRNE* has been previously reported only once [4]. We identified this mutation in a South Asian individual affected with CMS.

## Case presentation

**Fig. 1:**
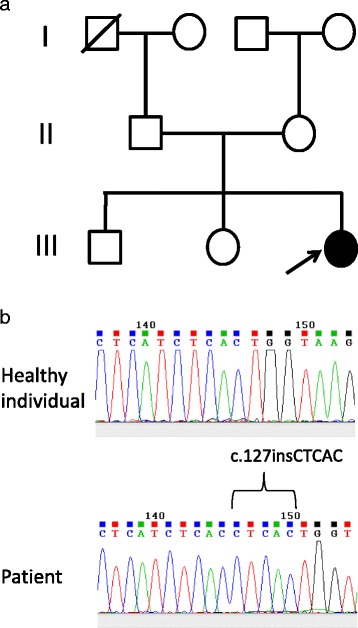
**a** Family tree of patient. Her paternal grandfather had died of malignancy. Arrow indicates proband. **b** Sequencing traces from a region within exon 2 of the *CHRNE* gene from a healthy individual and the patient. The 5-nucleotide insertion c.127insCTCAC is indicated

A 17-year-old Maldivian female domiciled in Sri Lanka presented with bilateral partial ptosis, fatigable proximal muscle weakness and slurring of speech noted since the age of 2 years. She recalls having difficulty in swallowing and being unable to run and play with her friends during her childhood and as she was growing up, requiring assistance to perform normal physical activities which her friends were able to do with ease, such as negotiating stairs. She particularly recalls being easily fatigued. She had been diagnosed with early childhood ‘autoimmune myasthenia gravis’ at the age of 4 years and commenced on pyridostigmine, which she had increased by herself over the years to her current regimen of 60 mg, five times a day as she had been experiencing greater fatigue with less frequent dosing. Her mother was uncertain of any definitive tests done, including acetylcholine receptor antibody status. The pyridostigmine provides her with symptomatic benefit but not completely, hence restricting her from activities that require rigorous effort. She cannot run, has difficulty negotiating stairs and rising from a seated position, and fatigues if she continues to speak at length. At times, particularly after a physically exhaustive day, she would experience blurring of vision and dysphagia, while even smiling would take much effort. Inter-current illnesses worsen her disabilities making her bed-bound and often requiring hospitalisation for specific and supportive treatment. Furthermore, she recalls that she takes longer than her friends to recover from similar illnesses. However, she has adapted within her limitations and remains independent in activities of daily living and has been able to continue with her aspired educational pathway, currently studying for a degree in forensic psychology. Her birth and past medical histories were otherwise unremarkable. There is no parental consanguinity or family history of muscle disorders (Fig. 1a).

On examination, she had a BMI of 18 kg/m^2^, bilateral fatigable partial ptosis, complete external ophthalmoplegia and fatigable proximal muscle weakness (MRC grade 4/5). Deep tendon reflexes were normal and there were no sensory deficits. Apart from spinal scoliosis, other systems examination was normal.

Haematological and biochemical investigations including full blood count, inflammatory markers, glucose, renal and liver function tests were normal. Serum lactate level was normal. Thyroid functions were within normal limits. Acetylcholine receptor antibodies and muscle specific kinase antibodies were not detected in serum. A decrement of >30 % was noted on repetitive nerve stimulation of facial nerves (right 2.61 to 1.39 mV and left 2.57 to 1.64 mV) and of radial nerves (right 0.55 to 0.36 mV and left 0.40 to 0.27 mV), which were done at the age of 17 years. Her DNA sequencing revealed an apparent duplication of nucleotide residues 183–187 within exon 2 of the AChR epsilon (ε) subunit gene (c.183_187dupCTCAC) (Fig. 1b).

## Conclusions

Genetic mutations responsible for CMSs have been identified in relation to proteins in the presynaptic, synaptic and postsynaptic compartments of the neuromuscular endplate. As expected, the frequency of particular gene mutations varies within different ethnic groups, but in most populations the most common form of CMS is due to a deficiency of acetylcholine receptors on the postsynaptic membrane [3]. These can result from recessive missense, nonsense, frameshift or splice site and promoter region mutations in any of the genes encoding the acetylcholine receptor subunits. However, a high frequency of mutations in the acetylcholine receptor ε subunit (CHRNE) compared with other subunits has been recognised and attributed to the phenotypic rescue by maintained low level expression of the foetal γ subunit that substitutes for the defective ε subunit, but cannot compensate for the other muscle acetylcholine receptor subunits. Our patient was diagnosed with c.183_187dupCTCAC within *CHRNE*, which is predicted to convert Leu–Asn–Glu codons to Pro–His–Stop at positions 63–65 of the protein p.(Leu63fs), resulting in truncation of the ε subunit in its extracellular domain [4]. This variant is SNP rs776927709, whose allele frequency count of 1.64728e-05 is low enough to be the cause of a rare recessively inherited genetic disease such as CMS. From the sequencing data presented here, this duplication is predicted to be homozygous. Sequence analysis of the parental DNA would provide confirmation but unfortunately parental DNA was not available. Therefore a heterozygous deletion with concomitant removal of one or both primer binding sites, which would produce the same chromatogram, cannot be excluded. Although our patient demonstrated the typical phenotype of *CHRNE* mutation CMS, a genetic diagnosis was confirmed only 15 years after onset, and a previous diagnosis of ‘autoimmune myasthenia gravis’ was revised precluding the risk of inadvertent immunotherapy. Furthermore, specific genetic diagnosis provided the option of adding 3,4 diaminopyridine and salbutamol when symptomatically required [2, 5].

In a review of patients attending a comprehensive neuromuscular clinic in India over a period of 8 years, 4.8 % of 314 myasthenic individuals were found to have CMS, but a genetic diagnosis had not been ascertained in them [6]. However, interestingly, certain founder mutations of CMS have been identified in individuals of Indian subcontinent origin [7, 8]. There was no family history of neuromuscular disorders in our patient, but unavailability of DNA from her parents precluded further evaluation of the origins of the identified mutation.

CMS can be clinically diagnosed on the basis of onset at birth to early childhood, fatigable weakness particularly of oculo-cranial muscles, a positive family history, and a decremental response on repetitive nerve stimulation. However, some patients may present later in life or the distribution of weakness may not be typical and a decremental response may not be easily evident, making differentiation from other neuromuscular disorders difficult. Some patients are mistakenly diagnosed with autoimmune myasthenia gravis mediated by antibodies against acetylcholine receptors or muscle specific tyrosine kinase, which are known to occur worldwide including in South Asia [9, 10], leading to inappropriate treatment with immunosuppressive drugs. Furthermore, drugs with mechanisms countering specific neuromuscular defects have been found to be effective in appropriate CMS subtypes, but harmful in other CMS subtypes with molecular defects inverse to the drug action [2]. Thus, a genetic diagnosis is important for diagnosis, treatment and prognostication of CMS.

Our case report adds to the number of CMS affected individuals identified with a rare mutation of the acetylcholine receptor ε subunit gene and highlights the importance of genetic diagnosis in CMS. It also highlights the occurrence of such mutations among South Asian populations.

## Abbreviations

BMI: Body mass index
CHRNE: Acetylcholine receptor ε subunit
CMS: Congenital myasthenic syndrome
DNA: Deoxyribonucleic acid
MRC: Medical Research Council
